# The effects of obesity on kidney function: a challenge for nephrologists

**DOI:** 10.1590/2175-8239-JBN-2019-0064

**Published:** 2019-05-30

**Authors:** Vera H. Koch

**Affiliations:** 1 Universidade de São Paulo Faculdade de Medicina Departamento de Pediatria São PauloSP Brasil Universidade de São Paulo, Faculdade de Medicina, Departamento de Pediatria, São Paulo, SP, Brasil.

Obesity is a chronic multifactorial disease stemmed from long-term positive energy balance that leads to excess adiposity and subsequent structural anomalies, physiological disorders, and functional impairment. Obesity increases the risk for other chronic conditions and has been associated with premature death.^1^

The global prevalence of obesity grew from less than 1% to 6-8% among boys and girls, 3% to 11% among men, and 6% to 15% among women between 1975 and 2016.^2^ Increased prevalence of obesity was followed by increased prevalence of hypertension, diabetes, and cardiovascular disease.^3^^,^^4^^,^^5^

The adverse effects of hypertension and peripheral insulin resistance coupled with systemic inflammation and dyslipidemia may trigger the development of chronic kidney disease (CKD).^6^ The combination of obesity and CKD has been a topic of debate in the literature.^7^^-^14

A recent meta-analysis looked into the findings from more than five million individuals from 40 countries and 63 general population cohorts, including patients with increased cardiovascular risk and patients with CKD. Studies published between 1970 and 2017 were analyzed for possible associations between measurements of adiposity, decreased estimated glomerular filtration rate (eGFR), and all-cause mortality. Long-term follow-up data revealed that individuals with a BMI greater than 30 kg/m^2^ belonging to general population cohorts were at significantly higher risk of suffering from eGFR decreases and showed a J-shaped association between BMI and death, with lower risk for individuals with a BMI of 25 kg/m^2^. In the cohorts with high cardiovascular risk and CKD, the association between an elevated BMI and lower eGFR was weaker than in the general population, but the J-shaped association between BMI and death with lower risk for individuals with a BMI between 25 and 30 kg/m^2^ persisted. The authors concluded that elevated BMI, waist circumference, and waist-height ratio were independent risk factor for eGFR declines and death in individuals with normal or decreased levels of eGFR.^15^

The deleterious effects of obesity on renal function may occur indirectly - via hypertension and/or diabetes mellitus - or directly by the production of adipokines, which trigger the onset of inflammation, oxidative stress, abnormal lipid metabolism, activation of the renin-angiotensin-aldosterone system, increased insulin production, and insulin resistance.^16^ These factors result in ectopic lipid accumulation in renal tissue, leading to functional and structural impairment of mesangial cells, podocytes, and the proximal tubule, culminating with glomerular hypertension, increased glomerular permeability, hyperfiltration, glomerulomegaly, albuminuria and even focal segmental glomerulosclerosis (FSGS) in some cases.^17^

Slow progression of non-nephrotic range proteinuria is the most common manifestation in obesity-related glomerulopathy (ORG). Massive proteinuria (> 5-10 g/day) may occur in some cases. Findings typical of nephrotic syndrome are usually absent in patients with nephrotic-range proteinuria. The harmful effects of obesity may combine with other renal conditions and add to the effects inherent to having a low number of nephrons, thereby accelerating progression to end-stage renal disease.^18^

Kidney biopsies of patients with ORG show more signs of glomerulopathy and fewer of glomerulosclerosis than the biopsies of patients with nephrotic syndrome. In the long run, patients with ORG treated with angiotensin-converting-enzyme (ACE) inhibitors or angiotensin II receptor blockers are less frequently affected by two-fold increases in serum creatinine levels and progression to end-stage renal disease than patients with nephrotic syndrome on immunosuppressants. Elevated serum creatinine at presentation and proteinuria are markers of progression to poor renal function in individuals with ORG.^19^

The BMI of pediatric patients changes as they grow, and should be interpreted as a function of the age and sex of the patient. Individuals with a BMI equal to or greater than the 85^th^ percentile *and* less than the 95^th^ percentile are overweight. Individuals with a BMI greater than the 95^th^ percentile for their age and sex are obese.^20^ The BMI cutoff points can accurately identify children at increased risk of becoming overweight and obese,^21^ and individuals at risk of developing cardiovascular risk factors (hypertension, dyslipidemia, and insulin resistance) in adult life.^22^^,^^23^

Differently from the adult population, there is no consensus over the definition of metabolic syndrome for pediatric patients. The definition used more frequently today comes from the International Diabetes Federation, which applies to patients aged 10 years or older and considers measurements of waist circumference (≥ 90^th^ percentile ot ≥ 94 cm for boys and ≥ 80 cm for girls), triglycerides ≥ 150 mg/dL, HDL-C (< 40 mg/dL for boys and < 50 mg/dL for girls), systolic/diastolic blood pressure ≥ 130 and/or 85 mmHg, and blood glucose ≥ 100mg/dL.^24^

The association between BMI and risk of developing end-stage renal disease was evaluated in 1.2 million 17-year-old adolescents followed for 30 years. The incidence rate of CKD in the period of the study was 2.87 cases per 100,000 person-years. When compared to adolescents with normal weight, overweight and obese adolescents had increased future risk for end-stage renal disease, with incidence rates of 6.08 and 13.40 cases per 100,000 person-years, respectively. In a multivariate model, overweightness and obesity were associated with development of all-cause end-stage renal disease (odds ratios of 3.00 and 6.89, respectively).^25^ Pre-bariatric surgery data of 242 adolescents included in the "Teen-LABS" trial showed that 14% had microalbuminuria; 3% had macroalbuminuria; 3% had eGFR < 60 mL/min/1.73m^2^; and 7.1% had eGFR > 150 mL/min/1.73m^2^. Increased BMI and HOMA-IR (Homeostasis Model Assessment for insulin resistance) index were significantly associated with lower eGFR.^26^ The patients were reviewed three years after surgery, revealing significant improvements in the mean eGFR, with an estimated gain of 3.9 mL/min/1.73m^2^ of eGFR for each 10-unit decrease of the BMI. Marked improvements were also seen in albuminuria levels in relation to preoperative values.^27^

This issue of the Brazilian Journal of Nephrology brings a cross-sectional study by Sawamura et al.^28^ in which 64 obese and overweight children and adolescents aged between five and 19 years were evaluated for frequency of albuminuria and its associations with severity of obesity, pubertal staging, morbidity, and eGFR. The mean age of the participants was 11.6 years and they were homogeneously distributed in relation to sex. Nearly half (45.3%) were prepubertal children. The high proportion of obese individuals (71.9%) in the series is noteworthy. The frequency and median value of albuminuria (> 30 mg/g) were 21.9% and 9.4 mg/g, respectively. The authors found no correlation between BMI, pubertal staging, insulin and HOMA-IR, or albuminuria levels and eGFR. The study did not show associations with other morbidities, with the exception of diastolic BP, which tended to higher values in individuals with microalbuminuria. The frequency of microalbuminuria found in this study was greater than the values reported in similar published studies. The disparity may stem from the different definitions used for microalbuminuria and the tests used to measure it. The absence of a control group in the study hinders the analysis of these variables.

In terms of the correlation between microalbuminuria and hypertension, Sawamura et al. used the 2004 Task Force report^29^ to categorize blood pressure levels. The categorization proposed in this report was recently updated^30^ with relevant changes in the parameters used to define hypertension in children and adolescents. The new definitions may potentially redefine the prevalence of hypertension in the general pediatric population,^31^ as well as in overweight and obese individuals.^32^

Another aspect to consider is the measurement of the eGFR in pediatric overweight and obese patients. The normalization of the eGFR to a standardized body surface area of 1.73 m^2^ was proposed to allow comparisons between the pediatric and adult populations.^33^ Since the BMI strongly correlates with body surface area, the adjustment for this parameter removes the effect of body weight in the GFR,^34^ thus underestimating the true GFR of individuals with a higher BMI and masking occurrences of hyperfiltration.^34^^,^^35^^,^^36^ A recent pediatric study showed that this issue may be overcome if the body surface area is calculated using the ideal instead of the actual weight.^37^ Another possibility revolves around the use of cystatin C, a marker deemed superior to the eGFR. Filler & Lepage, in a study enrolling children and adolescents with kidney conditions aged between one and 18 years, derived the following equation for the eGFR from serum cystatin C: log (GFR) = 1.962 + [1.123 * log (1 /Cystatin C)].^38^

The study by Sawamura et al. poses a challenge around the need to have overweight and obese children and adolescents followed by multiprofessional teams guided by prospective protocols to detect and manage the various potential complications arising from a clinical condition recently promoted to the status of global epidemics.
